# Exploring the intricate relationship between peptic ulcers and immunohematological responses: A narrative review

**DOI:** 10.1097/MD.0000000000042187

**Published:** 2025-04-11

**Authors:** Emmanuel Ifeanyi Obeagu, Getrude Uzoma Obeagu

**Affiliations:** a Department of Biomedical and Laboratory Science, Africa University, Mutare, Zimbabwe; b School of Nursing Science, Kampala International University, Kampala, Uganda.

**Keywords:** gastric mucosa, *Helicobacter pylori*, immune system, immunohematological responses, inflammation, pathogenesis, peptic ulcers

## Abstract

Peptic ulcers have long been a focus of medical research due to their significant impact on public health worldwide. Traditionally attributed to factors such as *Helicobacter pylori* infection and excessive gastric acid secretion, recent scientific endeavors have increasingly unveiled the pivotal role of immunohematological responses in the pathogenesis and clinical course of peptic ulcers. This review aims to synthesize and analyze the intricate relationship between peptic ulcers and immunohematological responses, shedding light on the complex interplay between the immune system and ulcer development, progression, and healing. Immunological factors, encompassing inflammatory mediators, immune cells, and the host response to *H pylori*, play a substantial role in the multifaceted landscape of peptic ulcers. Inflammation orchestrated by cytokines and chemokines derived from immune cells intricately contributes to mucosal damage and repair processes. Moreover, the chronic nature of *H pylori* infection triggers a cascade of immune responses, involving both innate and adaptive immunity, which significantly influences the course of ulceration. This paper consolidates current knowledge while highlighting the need for further research elucidating the intricate immunological pathways involved in peptic ulcer pathogenesis. The integration of immunology into the broader context of peptic ulcer disease presents opportunities for innovative therapeutic interventions aimed at modulating immune responses for improved clinical outcomes and enhanced patient care. Ultimately, unraveling the intricate relationship between peptic ulcers and immunohematological responses holds significant promise in advancing the understanding and management of this prevalent gastrointestinal disorder.

## 1. Introduction

Peptic ulcers, comprising both gastric and duodenal ulcers, have remained a significant health concern affecting millions of individuals worldwide. Historically, the etiology of peptic ulcers was predominantly linked to factors such as increased gastric acid secretion and the presence of *Helicobacter pylori* (*H pylori*) infection. However, contemporary research has illuminated a complex interplay between the immune system and the pathogenesis of peptic ulcers, underscoring the critical role of immunohematological responses in shaping the course of this gastrointestinal (GI) condition.^[1–3]^ While the association between *H pylori* infection and peptic ulcers is well-established, recent investigations have emphasized the profound impact of immunological factors on ulcer development, progression, and healing processes.^[4]^ The immune system’s intricate involvement in orchestrating inflammatory responses, regulating mucosal integrity, and responding to microbial challenges has unveiled new dimensions in our understanding of peptic ulcer disease (PUD).^[5]^

Peptic ulcers, characterized by mucosal erosions within the stomach or duodenum, represent a significant clinical challenge worldwide. While the pathogenesis of peptic ulcers has long been attributed to factors such as *H pylori* infection and nonsteroidal anti-inflammatory drugs use, emerging evidence suggests a complex interplay between these etiological factors and the host’s immunohematological responses. Immunohematological responses encompass a wide range of immune and hematological processes that influence the development, progression, and resolution of various diseases, including peptic ulcers. Immune cells, cytokines, chemokines, and other mediators of inflammation play pivotal roles in the pathophysiology of peptic ulcers by modulating mucosal defense mechanisms, promoting tissue damage, and influencing ulcer healing processes. Additionally, alterations in the hematological profile, such as changes in platelet function, coagulation cascade activation, and endothelial dysfunction, may contribute to ulcer formation and complications.^[2,3]^

The role of inflammation in PUD has gained increasing attention in recent years. Chronic inflammation, driven by factors like *H pylori* infection or nonsteroidal anti-inflammatory drugs, disrupts the delicate balance between mucosal defense and injury, leading to ulcer formation. Moreover, dysregulated immune responses, characterized by aberrant cytokine production and immune cell activation, can exacerbate mucosal damage and impair ulcer healing. Understanding the immunological mechanisms underlying PUD pathogenesis may unveil new therapeutic targets for modulating inflammation and promoting ulcer resolution. Furthermore, emerging evidence suggests bidirectional interactions between the gastrointestinal tract and the immune system, highlighting the importance of gut immunity in maintaining mucosal integrity and homeostasis. Disruption of this delicate equilibrium, whether due to dysbiosis, immune dysfunction, or environmental factors, can predispose individuals to peptic ulcer development. Conversely, peptic ulcers may exacerbate systemic inflammation and immune dysregulation through the release of inflammatory mediators into the circulation, potentially impacting distant organs and systems.^[4,5]^

This paper aims to explore and synthesize the evolving understanding of the relationship between peptic ulcers and immunohematological responses. It endeavors to elucidate the intricate mechanisms through which immune cells, inflammatory mediators, and host immune responses interact with microbial factors, environmental triggers, and genetic predispositions to influence the onset and clinical course of peptic ulcers. The inflammatory milieu within the gastric mucosa, orchestrated by a myriad of immune cells and signaling molecules, holds a pivotal role in perpetuating mucosal damage while concurrently initiating repair processes.^[6]^ Furthermore, the chronic nature of *H pylori* infection provokes a multifaceted immune response involving both innate and adaptive immunity, profoundly impacting the ulcerative process.^[7]^ Moreover, investigating the therapeutic implications of modulating immunohematological responses presents a promising avenue for tailored and effective treatment strategies for individuals afflicted by peptic ulcers.

## 2. Aim

The aim of this review is to comprehensively explore the intricate relationship between peptic ulcers and immunohematological responses.

## 3. Rationale

The rationale behind exploring the intricate relationship between peptic ulcers and immunohematological responses lies in the growing recognition of the multifaceted nature of PUD pathogenesis. While factors such as *H pylori* infection and nonsteroidal anti-inflammatory drugs have traditionally been considered key contributors to ulcer formation, emerging evidence suggests that immunological and hematological processes play significant roles in modulating disease progression and outcomes. Understanding the immunohematological responses associated with peptic ulcers is crucial for several reasons. Firstly, it offers insights into the complex interplay between the host immune system, mucosal defense mechanisms, and ulcerogenic factors, providing a more holistic understanding of PUD pathophysiology. Secondly, elucidating the immunopathogenesis of peptic ulcers may identify novel biomarkers for disease diagnosis, prognosis, and treatment response assessment, thereby enhancing clinical management strategies. Furthermore, exploring the immunohematological aspects of peptic ulcers may uncover potential therapeutic targets for intervention. By targeting immune-mediated pathways or modulating hematological processes implicated in ulcer formation and healing, novel treatment approaches could be developed to improve patient outcomes and reduce the burden of PUD-related complications. Moreover, considering the bidirectional interactions between the GI tract and the immune system, understanding how peptic ulcers influence systemic immune responses and hematological parameters may have broader implications for the management of GI and systemic diseases. Insights gained from studying the immunohematological aspects of PUD could potentially inform therapeutic strategies for related conditions characterized by mucosal inflammation, such as inflammatory bowel disease or gastroesophageal reflux disease (GERD).

## 4. Review methodology

### 4.1. Literature search strategy

A comprehensive literature search was conducted using electronic databases such as PubMed, MEDLINE, Embase, and Google Scholar. Keywords and Medical Subject Headings (MeSH) terms related to peptic ulcers, immunohematological responses, inflammation, immune system, hematological parameters, and associated concepts were used in various combinations to identify relevant studies. Filters for publication date, language, and study type were applied as appropriate to ensure the inclusion of the most recent and pertinent research articles, reviews, and meta-analyses.

### 4.2. Study selection criteria

- Inclusion criteria
- Studies investigating the relationship between peptic ulcers and immunohematological responses.
- Original research articles, review papers, meta-analyses, and systematic reviews.

Studies published in peer-reviewed journals.

- Exclusion criteria
- Studies unrelated to peptic ulcers or immunohematological responses.
- Case reports, editorials, letters, and conference abstracts.

### 4.3. Data extraction

Relevant data from selected studies were extracted and organized using a standardized data extraction form. Extracted data included study characteristics (author, year of publication, study design), participant demographics, interventions or exposures, outcomes of interest (immunohematological parameters, ulcer severity, healing rates), and key findings.

### 4.4. Quality assessment

The quality and risk of bias of included studies were assessed using appropriate tools such as the Newcastle-Ottawa Scale for observational studies or the Cochrane risk of bias tool for randomized controlled trials. Studies were evaluated based on criteria such as study design, sample size, methodology, participant selection, outcome measurement, and potential sources of bias.

### 4.5. Data synthesis and analysis

Extracted data were synthesized narratively to provide an overview of the current evidence regarding the relationship between peptic ulcers and immunohematological responses. Findings from individual studies were synthesized to identify common themes, patterns, and discrepancies. Where appropriate, quantitative data were pooled for meta-analysis to generate summary estimates of effect sizes and assess heterogeneity across studies. Conflicting findings were explicitly addressed by analyzing study methodologies, sample populations, and potential biases. For instance, variations in reported cytokine levels or immune cell responses were attributed to differences in patient demographics, disease stages, and experimental protocols.

## 5. Inflammatory molecules and cytokines

Inflammatory mediators and cytokines play pivotal roles in the body’s immune response, particularly in the context of inflammation, infection, and tissue repair. They are signaling molecules produced by various cells, including immune cells, and serve as messengers that regulate immune and inflammatory processes.^[8]^ Released by mast cells and basophils, histamine increases blood vessel permeability, leading to swelling and redness.^[9]^ It also causes smooth muscle contraction. These lipid compounds modulate inflammation, pain, and fever. They are produced by cyclooxygenase enzymes and are involved in vasodilation, fever induction, and sensitizing nerves to pain.^[10]^ Similar to prostaglandins, leukotrienes are lipid mediators that contribute to inflammation, particularly in allergic and asthmatic reactions. They cause bronchoconstriction and increased mucus production.^[11]^ This peptide causes blood vessel dilation, increases vascular permeability, and contributes to pain sensation. A group of proteins that work together to promote inflammation, enhance phagocytosis, and contribute to the destruction of pathogens.

Cytokines are small proteins secreted by immune cells that regulate immune responses and mediate communication between cells.^[12]^ ILs are a diverse group of cytokines involved in communication between immune cells. For example, IL-1 and IL-6 promote inflammation, while IL-2 stimulates the proliferation of T cells. A potent pro-inflammatory cytokine involved in the regulation of immune cells and inflammation. It plays a critical role in several inflammatory diseases. These cytokines help the body defend against viruses and regulate the immune response.^[13]^ They are used in the treatment of viral infections and some cancers. These cytokines guide the migration of immune cells to sites of infection or injury by inducing chemotaxis. They are involved in the trafficking and positioning of immune cells within tissues.^[13]^

## 6. Immune cells and their effector functions

The immune system is a complex network of cells, tissues, and organs that work together to defend the body against pathogens, such as bacteria, viruses, fungi, and parasites, as well as against abnormal cells (like cancer cells). Immune cells play diverse roles and can be broadly categorized into 2 main types: innate immune cells and adaptive immune cells. Neutrophils are the most abundant type of white blood cells. They are rapid responders to infection and are highly phagocytic, meaning they engulf and destroy pathogens.^[14]^ Macrophages are phagocytic cells that patrol tissues, engulf pathogens, and cellular debris. They play a crucial role in initiating and regulating the immune response.^[15]^ Dendritic cells are essential for presenting antigens to T cells, thereby initiating adaptive immune responses. They are like “sentinels” that detect pathogens and activate the adaptive immune system.^[16]^ Natural killer cells provide rapid responses against virus-infected cells and cancer cells by releasing cytotoxic molecules that induce cell death.^[17]^ Basophils, Eosinophils, and Mast Cells are involved in allergic responses and defense against parasites. Mast cells, in particular, release histamine and other inflammatory mediators.

T Lymphocytes (T Cells)mature in the thymus and play various roles. Helper T cells coordinate the immune response by secreting cytokines that activate other immune cells.^[18]^ Cytotoxic T cells (Tc cells) recognize and kill virus-infected or abnormal cells directly. Regulatory T cells (Treg cells) suppress the immune response to prevent autoimmunity. B Lymphocytes (B Cells) produce antibodies (immunoglobulins) that recognize and neutralize pathogens. Memory B cells help provide long-term immunity upon reexposure to the same pathogen. Engulfment and destruction of pathogens or foreign particles by phagocytic cells like neutrophils, macrophages, and dendritic cells. The ability of certain immune cells, such as natural killer cells and cytotoxic T cells, to induce cell death in infected or abnormal cells. Immune cells release cytokines that regulate the immune response, coordinate cell-to-cell communication, and modulate inflammation. B cells produce antibodies that recognize and bind to specific antigens on pathogens, marking them for destruction by other immune cells or neutralizing their harmful effects. Treg cells and other regulatory mechanisms help maintain immune tolerance, preventing the immune system from attacking healthy cells and tissues. Effector functions vary among different immune cells and are coordinated to provide a robust and effective defense against diverse threats to the body.^[19]^ Interactions and communication between these cells are tightly regulated to ensure an appropriate immune response while avoiding self-damage.

## 7. H pylori-induced immune responses

*H pylori* is a bacterial pathogen that colonizes the human stomach and is a major cause of various gastric conditions, including chronic gastritis, peptic ulcers, and gastric cancer.^[20]^ *H pylori* infection triggers the activation of innate immune cells like neutrophils, macrophages, dendritic cells, and natural killer cells in the stomach lining. Innate immune cells release pro-inflammatory cytokines (such as interleukin-1β, interleukin-8, and tumor necrosis factor-alpha [TNF-α]) and chemokines in response to *H pylori*, promoting inflammation and recruiting additional immune cells to the infection site.^[21]^ Pattern recognition receptors recognize specific components of *H pylori* (such as lipopolysaccharides and flagellin), initiating the innate immune response.

*H pylori* induces a robust T-cell response, particularly CD4 + T cells (helper T cells), which release cytokines like interferon-gamma and interleukins. These cells play a critical role in the immune response against the bacterium.^[18]^ B cells are activated, leading to the production of antibodies, primarily IgG and IgA, against *H pylori* antigens. However, the bacterium has evolved mechanisms to evade the immune response, such as altering its surface antigens. *H pylori* employs various strategies to evade the host immune response and establish chronic infection. These include antigenic variation, modulation of host immune cell functions, and influencing Treg cells to create an immune-suppressive environment. Prolonged *H pylori* infection can lead to chronic inflammation of the gastric mucosa due to continuous immune activation.^[22]^ This persistent inflammation can contribute to the development of gastritis, peptic ulcers, and may increase the risk of gastric cancer over time.

## 8. Mucosal barrier integrity and immunological factors

Maintaining mucosal barrier integrity is crucial for the immune system’s function in protecting against pathogens and preventing inflammatory responses to harmless antigens.^[23]^ The mucosal surfaces, such as those in the GI, respiratory, and urogenital tracts, are in constant contact with the external environment and face challenges from potential pathogens, antigens, and commensal microorganisms. Mucosal surfaces are lined with epithelial cells that act as a physical barrier preventing the entry of pathogens. Tight junctions between epithelial cells regulate permeability. Mucins produced by goblet cells form a mucus layer that covers and protects the epithelial surface. Mucus acts as a physical barrier and traps pathogens, preventing their adherence to and invasion of epithelial cells.^[23]^ Secreted by epithelial cells, Antimicrobial Peptides like defensins and cathelicidins have antimicrobial properties, directly killing or inhibiting the growth of pathogens.^[24]^ Produced by plasma cells in mucosal tissues, Secretory Immunoglobulin A (sIgA) is the predominant immunoglobulin at mucosal surfaces.^[25]^ It helps neutralize and eliminate pathogens, preventing their attachment and entry into tissues.

## 9. Immunological factors at mucosal surfaces

Mucosal-associated lymphoid tissue are specialized immune structures like Peyer’s patches in the intestine and tonsils in the respiratory tract house immune cells. These structures sample antigens and generate immune responses.^[26]^ Present in mucosal tissues, dendritic cells capture antigens, process them, and present them to T cells, initiating adaptive immune responses. Mucosal surfaces contain various subsets of T and B cells that participate in immune surveillance, antibody production, and regulatory functions. For instance, Treg cells help maintain immune tolerance to harmless antigens.^[27]^ Cytokines and chemokines regulate immune cell activation, recruitment, and function at mucosal sites. They modulate inflammation, immune responses, and tissue repair.^[18]^

A robust mucosal barrier prevents the entry and colonization of pathogens, reducing the risk of infections.^[24]^ The mucosal immune system must distinguish between harmful pathogens and harmless antigens to avoid unnecessary immune responses or inflammation against harmless substances, such as food or commensal microbes. Proper functioning of mucosal barriers and immune factors helps maintain a balance between protection from pathogens and tolerance to innocuous antigens, contributing to overall health. Impairment of mucosal barrier integrity or dysregulation of immune responses at mucosal surfaces can lead to inflammatory bowel diseases, allergic reactions, autoimmune disorders, and increased susceptibility to infections.

## 10. Immunomodulatory therapies and future perspectives

Immunomodulatory therapies refer to treatments that modify or regulate the immune system’s response, either by enhancing or suppressing it. These therapies have diverse applications in treating various diseases, including autoimmune disorders, inflammatory conditions, cancer, and infectious diseases. Monoclonal antibodies are engineered antibodies that target specific molecules or cells involved in the immune response. They are used in conditions like rheumatoid arthritis, inflammatory bowel disease, and certain cancers.^[28]^ Some diseases involve dysregulated cytokine signaling. Therapies that block or mimic cytokines (e.g., TNF-α inhibitors) are used in autoimmune diseases like rheumatoid arthritis and psoriasis.^[18]^ Corticosteroids are potent anti-inflammatory drugs suppress the immune system and are used in various autoimmune and inflammatory conditions but may have significant side effects with prolonged use.^[29]^ Immunosuppressants are drugs like methotrexate, azathioprine, and cyclosporine are used to suppress the immune system in autoimmune diseases and following organ transplantation to prevent rejection.

These drugs block inhibitory signals in T cells, allowing the immune system to recognize and attack cancer cells. Checkpoint inhibitors like PD-1 and CTLA-4 inhibitors have revolutionized cancer treatment but can lead to immune-related adverse effects.^[30]^ Cancer Immunotherapies harness the immune system to target cancer cells, including adoptive cell transfer (CAR-T cell therapy), tumor-infiltrating lymphocytes, and therapeutic vaccines. Allergy Immunotherapy involves exposing individuals to small amounts of allergens to desensitize their immune response, reducing allergic reactions.

## 11. Linking immune mechanisms to therapeutic strategies in PUD

The intricate relationship between immune mechanisms and peptic ulcers provides a foundation for developing targeted therapeutic strategies.

### 11.1. Cytokine responses and anti-inflammatory therapies

Pro-inflammatory cytokines such as interleukin-1β (IL-1β), TNF-α, and interleukin-8 (IL-8) are central to the inflammatory cascade in peptic ulcers. These cytokines exacerbate tissue damage by recruiting neutrophils, amplifying oxidative stress, and disrupting mucosal integrity. Drugs targeting specific cytokines, such as TNF-α inhibitors (e.g., infliximab), could mitigate inflammation and prevent further mucosal injury. Blocking IL-8 or its receptors may reduce neutrophil recruitment and subsequent oxidative damage, promoting healing.^[29]^

### 11.2. Immune cell roles and immune modulation

Immune cells, including macrophages, neutrophils, and T-helper (Th) cells, are key players in the immune response to peptic ulcers. Macrophages contribute to tissue repair but also release inflammatory mediators that can prolong damage. Th1 and Th17 Cells drive the chronic inflammatory response, while Treg cells attempt to dampen inflammation but may be overwhelmed. Therapies that boost Treg function or numbers could help restore immune balance and reduce inflammation. Targeting neutrophil activation pathways or their lifespan (e.g., using CXCR inhibitors) may limit collateral damage while preserving essential defense mechanisms.^[30]^

### 11.3. Mucosal immunity and protective strategies

The gastric mucosa relies on a delicate balance between protective factors (e.g., mucus, bicarbonate) and immune responses to maintain integrity. Disruptions in mucosal immunity, such as weakened barrier function or impaired immune surveillance, can exacerbate ulcer formation. Specific strains, such as *Lactobacillus* and *Bifidobacterium*, enhance mucosal immunity by reducing *H pylori* colonization and modulating inflammatory responses. Compounds like rebamipide can strengthen the mucosal barrier by promoting mucus production and reducing oxidative stress.

### 11.4. H pylori and immunotherapy

*H pylori*, a key pathogen in PUD, manipulates the immune system by evading phagocytosis and dampening T-cell activation. This persistent infection triggers chronic inflammation, promoting ulcerogenesis. Developing vaccines targeting *H pylori* antigens could prevent infection and reduce the incidence of ulcers. Using adjuvants to enhance the immune response against *H pylori* may improve eradication rates.^[30]^

### 11.5. Chronic inflammation and systemic immune modulators

Chronic inflammation in peptic ulcers can lead to systemic immune dysregulation, including elevated circulating cytokines and leukocyte activation. Addressing this systemic impact is crucial for long-term management. Anti-inflammatory biologics like monoclonal antibodies against specific cytokines (e.g., IL-6) could mitigate systemic inflammation. Therapies that modulate systemic immune responses, such as low-dose immunosuppressants or immune checkpoint inhibitors, may help restore balance.^[29]^

## 12. Future perspectives

Advancements in personalized medicine aim to tailor immunomodulatory therapies based on an individual’s immune profile, genetic makeup, and disease characteristics for more targeted and effective treatments. Technologies like CRISPR/Cas9 offer potential in modifying immune cells ex vivo for targeted therapies in autoimmune diseases, cancer, and genetic immune disorders. Nanoparticle-based therapies and drug delivery systems hold promise in precisely targeting immune cells or tissues, improving therapeutic efficacy, and reducing side effects. Future strategies may involve combining different immunomodulatory agents, such as checkpoint inhibitors with other immune-based therapies, to enhance treatment outcomes and reduce resistance.

## 13. Clinical and health implications

*Diagnostic Advances*: Incorporating immunohematological markers into diagnostic algorithms may enhance the accuracy and precision of peptic ulcer detection. Utilizing specific immune cell profiles, cytokine signatures, or serological markers associated with immune responses can aid in early and precise diagnosis, allowing for timely intervention and improved clinical outcomes.

*Tailored Therapeutic Approaches*: Understanding the immunohematological intricacies in peptic ulcer pathogenesis opens avenues for personalized therapeutic interventions. Targeting specific immune pathways or utilizing immunomodulatory agents may pave the way for tailored treatment strategies. Immunotherapies aimed at modulating immune responses could complement traditional treatments, potentially enhancing healing rates and reducing recurrence.

*Reduced Antibiotic Resistance and Treatment Optimization*: Insights into the immunological aspects of *H pylori* infection may lead to strategies that minimize antibiotic resistance. Innovative approaches targeting immune responses against *H pylori* could reduce reliance on antibiotics, potentially optimizing treatment efficacy and minimizing side effects associated with antimicrobial therapy.

*Preventive Strategies:* Comprehensive understanding of immunohematological responses in peptic ulcers may guide the development of preventive strategies. Vaccines targeting specific antigens or immune-stimulating agents aimed at bolstering mucosal immunity could potentially prevent *H pylori* infection or mitigate its detrimental effects, thereby reducing the risk of ulcer development.

*Improved Patient Management and Prognosis:* Integrating immunological assessments into patient management protocols could refine prognostic evaluations. Monitoring immune markers or cytokine profiles may aid in predicting ulcer severity, response to treatment, and risk of recurrence, thereby facilitating more personalized and precise patient care.

*Enhanced Patient Education and Lifestyle Modifications*: Increased awareness of the role of immunohematological factors in peptic ulcers could empower patients to make informed lifestyle choices. Understanding how diet, stress, and other factors impact immune responses may motivate individuals to adopt healthier lifestyles, potentially reducing the risk of ulcer development or exacerbation.

*Research and Development of Novel Therapeutics*: Continued exploration of the immunological landscape of peptic ulcers is essential for the development of innovative therapeutics. Research in immunotherapy, immune-modulating drugs, and targeted interventions based on specific immunological pathways holds promise for advancing ulcer treatment and improving overall patient care.

## 14. Recommendations

Further research should focus on identifying specific immunological biomarkers that correlate with peptic ulcer development, severity, and healing. Validating these markers could enhance diagnostic accuracy, enabling clinicians to promptly identify at-risk individuals and initiate targeted interventions. Conducting clinical trials to evaluate the efficacy and safety of personalized immunomodulatory therapies is crucial. Investigating the use of targeted immunotherapies tailored to individual immune profiles or specific immunological pathways could lead to more effective and tailored treatment options. Encourage the integration of immunomodulatory agents with conventional therapies (such as proton pump inhibitors and antibiotics) to explore synergistic effects. Comprehensive treatment protocols that combine immunological interventions with standard therapies may enhance healing rates and reduce the likelihood of ulcer recurrence.

Invest in the development and evaluation of vaccines targeting *H pylori*-specific antigens. Immunization strategies aimed at preventing *H pylori* infection or reducing its virulence could be pivotal in preventing ulcer development and decreasing the burden of associated complications. Conduct longitudinal studies to assess changes in immune profiles throughout the course of PUD. Long-term monitoring of immune markers in patients undergoing treatment can provide valuable insights into disease progression, treatment response, and recurrence risk, aiding in personalized patient management.

Develop educational programs to familiarize healthcare professionals with the evolving understanding of immunohematological responses in peptic ulcers. Continuous medical education focused on the intersection of immunology and gastroenterology would enable clinicians to integrate immunological insights into their practice effectively. Launch public awareness campaigns to educate individuals about the impact of lifestyle choices on immune responses and peptic ulcer development. Promoting healthy dietary habits, stress management, and avoidance of risk factors could potentially reduce the incidence and severity of peptic ulcers. Encourage collaboration between gastroenterologists, immunologists, microbiologists, and pharmacologists to foster multidisciplinary research initiatives. Collaborative efforts can facilitate a more holistic understanding of the immunological mechanisms underlying peptic ulcers, leading to innovative discoveries and therapeutic advancements.

Ensure stringent ethical oversight in clinical trials exploring immunotherapy for peptic ulcers. Emphasize patient safety, informed consent, and rigorous monitoring of potential side effects associated with novel immunomodulatory agents or therapies. Advocate for the integration of immunological insights into global health policies concerning peptic ulcer prevention, diagnosis, and treatment. Collaborate with health organizations to incorporate immunological considerations into standardized guidelines for managing PUD.

## 15. Conclusion

The exploration of immunohematological responses in the context of peptic ulcers has unveiled a new frontier in understanding the multifaceted nature of this prevalent GI disorder. Traditionally viewed through the lens of acid secretion and microbial infection, recent advancements highlight the pivotal role played by the immune system in the pathogenesis, progression, and management of peptic ulcers. The intricate interplay between inflammatory mediators, immune cells, and the host response to *H pylori* infection significantly influences the delicate balance between mucosal damage and repair mechanisms. The evolving landscape of immunomodulatory therapies, personalized treatment approaches based on immune profiles, and the potential for immunization strategies against *H pylori* offer promising avenues for enhanced patient care. Furthermore, integrating immunological insights into clinical practice could refine diagnostic accuracy, optimize treatment efficacy, and improve prognostic evaluations. In essence, acknowledging the intricate relationship between peptic ulcers and immunohematological responses paves the way for a holistic approach to managing this condition.

## Author contributions

**Conceptualization:** Emmanuel Ifeanyi Obeagu.

**Methodology:** Emmanuel Ifeanyi Obeagu, Getrude Uzoma Obeagu.

**Resources:** Emmanuel Ifeanyi Obeagu.

**Supervision:** Emmanuel Ifeanyi Obeagu.

**Validation:** Emmanuel Ifeanyi Obeagu.

**Visualization:** Emmanuel Ifeanyi Obeagu.

**Writing – original draft:** Emmanuel Ifeanyi Obeagu, Getrude Uzoma Obeagu.

**Writing – review & editing:** Emmanuel Ifeanyi Obeagu, Getrude Uzoma Obeagu.
